# Effect of Study-Duration and Time of Day on Multichannel Sleep Study Findings in Former Preterm Infants

**DOI:** 10.3390/children12010074

**Published:** 2025-01-08

**Authors:** Allison Sadowski, Naveed Hussain, Leonard I. Eisenfeld, Mariann Pappagallo, Janet Schwenn, Ted S. Rosenkrantz

**Affiliations:** 1 Connecticut Children’s Medical Center—Hartford, 282 Washington Street, Hartford, CT 06106, USA; asadowski02@connecticutchildrens.org (A.S.); leisenf@connecticutchildrens.org (L.I.E.); pappagallo@uchc.edu (M.P.); rosenkrant@uchc.edu (T.S.R.); 2 Division of Neonatology, Department of Pediatrics, University of Connecticut School of Medicine, Hartford, CT 06032, USA; 3 Department of Nursing, UCONN Health, Farmington, CT 06030, USA; janet2schwenn@gmail.com

**Keywords:** pneumogram, sleep study, polysomnography, infant, premature, multi-channel recording, apnea, bradycardia, desaturations, breathing abnormalities

## Abstract

Background/Objectives: Determine the appropriate duration for multichannel sleep studies in former preterm infants with cardio-respiratory events beyond term equivalent age. Hypothesis: A sleep study of 10 h will provide equivalent information compared to a 20-h study to detect significant cardio-respiratory abnormalities in this population. Methods: Single-center retrospective study of 50 infants with 20-h sleep study. Studies were evaluated for periodic breathing, obstructive, central, mixed apnea, desaturations, and bradycardia. Each study was partitioned into two 10-h epochs, compared to one another and the 20-h study. Results: Differences were detected at the level of individual sleep studies when each epoch was compared to each other and a total 20-h study. 10-h study missed 17–31% of breathing abnormalities detected over 20 h adjusted for study time. Group analysis showed no statistical difference in the number and duration of events between epochs. Conclusions: A 20-h sleep study improves the detection of breathing abnormalities missed with a 10-h study.

## 1. Introduction

Apnea and periodic breathing, associated with bradycardia or oxygen desaturation, are common problems in preterm infants. There is a lack of clarity regarding the definition of apnea of prematurity (AOP) [1]. However, the commonly used definition is a pause in breathing of ≥20 s or <20 s if associated with significant bradycardia with or without oxygen desaturation [2]. AOP results from an immature respiratory center in premature infants [3,4,5]. Specifically, it is related to immature neurochemical regulation of central and peripheral breathing responses to hypoxia and hypercapnia with possible influences of neurotransmitters such as GABA [6,7]. Long-term complications of recurrent episodes of apnea are of concern but remain to be thoroughly evaluated [7,8,9,10]. Darnell et al. have shown that repeated AOP episodes can lead to inflammation in the brain with associated injury in a rat model [11]. Janvier et al. demonstrated a significant association between the total number of days with apnea noted in the hospital and worse neurodevelopment outcomes [10]. Animal models have demonstrated that increased episodes of hypoxia secondary to apnea are associated with decreased myelinogenesis and delayed axonal maturation [12]. Although older clinical studies have minimized the effect of persistent apneic episodes in outcomes of preterm infants, newer reports are suggesting that apnea occurring in the neonatal period should be diligently identified and treated adequately to decrease potential future adverse effects [13,14].

Most premature infants resolve AOP by term corrected age (38–40 weeks) but a subset of premature infants have unresolved breathing abnormalities beyond the expected resolution time [2]. These infants may have clinically significant and persistent periodic breathing or apnea associated with bradycardia and/or oxygen desaturation that delays their discharge from the NICU. There is another subset of infants in the NICU who are born at term gestation but have clinically significant apnea and/or periodic breathing that requires evaluation before they can be safely discharged home. It is essential to distinguish the cause of breathing abnormalities, hypoxia, and/or bradycardia before a proper management plan can be developed for these infants [7]. One objective and definitive evaluation method of these infants is a multichannel sleep study with simultaneous recordings of nasal airflow, chest wall movement, infant activity, heart rate, and oxygen saturation [15,16,17]. Data obtained from neonatal multichannel sleep studies have been used to determine if the observed events are due to artifacts, immature central respiratory control, airway obstruction, or a combination of factors [18]. Understanding the cause of these events could guide proper treatment and develop a safe discharge plan. Infants experiencing hypoxia/bradycardia due to immature respiratory control (apnea or periodic breathing) benefit from caffeine therapy. They can be discharged home on caffeine and a home monitor with regular follow-up. Infants having obstructive events may need an evaluation of airway anatomy and function, gastroesophageal reflux, or neurological problems. A normal multichannel sleep study helps to exclude respiratory events as the cause of the infant’s cardio-respiratory events. At our center, the entire study time for a multichannel sleep study is 20 h as we perform the study while the infant is undergoing a pH/impedance study for gastroesophageal reflux. The standard of care for pH/impedance studies is 20 h. However, no standard time duration is recommended in published literature for a neonatal multichannel sleep study. Multichannel sleep studies require significant setup, recording, scoring, and analysis resources. This translates into a considerable investment of time. While a shorter study time could reduce workload with cost savings, it is essential to know what an adequate amount of study time is to detect abnormalities. Realizing there is some degree of variability in the study results, it is also essential to know if there are significant diurnal effects. We hypothesized that a multichannel sleep study duration of 10 h at any time is adequate and equivalent to a 20-h study period to evaluate respiratory abnormalities in former preterm infants in the NICU. Our specific aims were (i) to determine if a shorter multichannel sleep study could be done without affecting the accuracy of the study and (ii) to as determine if diurnal variations would affect the results of the study.

## 2. Materials and Methods

### 2.1. Study Design

A retrospective study used a convenience sample of 50 consecutive preterm infants who underwent multichannel sleep studies between January 2012 and January 2016 at Connecticut Children’s Medical Center (CCMC) NICU in Farmington, CT. The 20-h-Multichannel Sleep Studies were done to develop a management plan in infants at or beyond term post-menstrual age with clinically significant cardio-respiratory events. CCMC IRB approved the retrospective collection and analysis of data for this study.

### 2.2. Data Collection

Data collected for this study included patient demographic information, birth date, indication for the test, infant weight, and postmenstrual age at the time of the study, as well as medications in use at the time of the study. Data on co-morbid conditions included diagnoses of intraventricular hemorrhage (IVH), periventricular leukomalacia (PVL), bronchopulmonary dysplasia (BPD), airway problems, hypoxic-ischemic encephalopathy, and necrotizing enterocolitis (NEC). Recordings from the 20-h sleep studies were evaluated as detailed below.

### 2.3. Multichannel Sleep-Study Method

For the multichannel sleep study, physiologic data were initially recorded on a laptop computer at the bedside through a Philips Respironics Alice 6 LDx interphase using multiple input channels. Five data channels were gathered and analyzed using proprietary software (Alice 5^®^ software). Channel 1—Nasal airflow: nasal prongs were used with thermistors to detect airflow. Channel 2—Chest movements related to respiration were recorded using impedance sensors placed within a belt snugly wrapped around the middle part of the chest. Channel 3—EKG leads were used for recording cardiac activity. Channel 4—Oxygenation data were recorded using a pulse oximeter wrapped around the foot. Channel 5—an ‘actimeter’ on the lower leg was used to record activity to help distinguish awake and sleeping times. No EEG recordings were done. This Multichannel Sleep Study methodology was similar to the one Graff et al. reported [17]. All measurements were made with a dedicated laptop at the bedside while the infant was managed with the usual routine of feeding, sleeping, and lying in bed, as positioning has not been shown to affect the results. All infants were fed by bottle or breast. A 20-h recording was done for all patients studied, and in patients suspected of GER, an additional data channel recorded information from a pH-impedance probe. For infants with pH-impedance recording, a dedicated bedside nurse ensured documentation of all infant activity during the study, including feeding times and any clinically significant events observed. After the conclusion of the study, the data from the laptop were transferred to a desktop computer with the Alice^®^ software using a SIM card.

### 2.4. Multichannel Sleep Study Evaluation

The study was initially evaluated and coded using Alice^®^, an automated software analysis. Alice 5^®^ software uses a well-known and validated algorithm to evaluate neonatal cardiorespiratory problems. A trained respiratory therapist with >15 years of experience reviewed the automated analysis for accuracy and artifact before the final result output was generated. Apnea, both central and obstructive, periodic breathing, isolated hypoxia, and isolated bradycardia were identified from the sleep portion of the recording. Trained and experienced personnel (JS and TSR) independently analyzed the analog patterns recorded and confirmed that the events coded by the computer software were appropriate. After confirmation of the events was finalized, the Alice^®^ software was used to generate a detailed report of the study. This report included the number and duration of periodic breathing events, obstructive, central, and mixed apnea events, oxygen desaturation episodes, and bradycardic events, as well as the association of apnea and periodic breathing with oxygen desaturation and bradycardia. After completing the 20-h evaluation, the same study was recoded into two equal epochs. The first epoch included recordings from 12 PM to 10 PM (epoch A or Afternoon epoch) and a 10 pm–8 am recording (epoch B or Bedtime epoch). Although the light level in the NICU is not standardized, the unit is quieter during the night shift with lower light levels. Since these infants were studied close to their discharge date, the effect of prone/supine positioning was not evaluated, as all infants slept in the supine position per the AAP Safe-Sleep policy.

### 2.5. Definitions

The following definitions were used to evaluate the studies. Apnea was defined as a pause in breathing of ≥20 s duration or a pause in breathing of <20-s duration if associated with significant bradycardia with or without oxygen desaturation [2]. Bradycardia was defined as a recorded drop in heart rate below 80 beats/minute for >10 s. Desaturation was defined as a recorded drop in saturations below 90% for >10 s [19]. Periodic Breathing was defined as three or more episodes of central apnea lasting at least 3 s, separated by no more than 20 s of normal breathing. The distinctions of central apnea, obstructive apnea, and mixed apnea were based on changes in the recordings of respiratory events from the nasal thermistor prongs and the chest impedance leads. Sleep study results were given an operational definition of ‘normal’ if the following criteria were met: (a) absence of central apnea, (b) <5% time in periodic breathing, (c) no change in oxygen desaturation during the periodic breathing, and (d) absence of obstructive or mixed apnea with associated desaturations or bradycardia. The remainder of the studies were ‘abnormal’ if they had one or more of the four criteria present (apnea, >5% periodic breathing, oxygen desaturation during periodic breathing, or bradycardia). The reason for an ‘abnormal’ study was also noted and evaluated.

The time of day of occurrence of the critical event(s) from a multichannel Multichannel Sleep Study that led to the final diagnosis of ‘normal’ or ‘abnormal’ sleep study were also analyzed within the two epochs (A—Afternoon epoch or B—Bedtime epoch).

### 2.6. Statistical Analysis

The characteristics of the infants and their 20-h Multichannel Sleep Study that were categorized as normal were compared to those that were abnormal. Unpaired T-tests or Chi-Square tests were done for univariate analysis. The morbidity correlates of the normal and abnormal studies were also analyzed. For comparison of the occurrence of cardiorespiratory events that occurred in the two 10-h epochs (A—Afternoon; B—Bedtime), paired *t*-tests were done. The comparisons between the two 10-h epochs and the complete 20-h study were done using repeated measures ANOVA. Sensitivity, specificity, and predictive values of clinical symptoms with normal or abnormal 20-h multichannel sleep study results were also evaluated.

## 3. Results

### 3.1. Patient Characteristics

Patient characteristics are shown in Table 1. Comparing those with Abnormal sleep study (*n* = 41) vs those with normal study (*n* = 9), there were no differences in gestational age at birth or birthweight or the corrected gestational age or weight at the time of the study. The mean age of the infant at the time of the study was 39.6 weeks, with a range of 38.0–41.2 weeks. There also were no differences in infant sex, race, birth sequence, or mode and place of delivery. Abnormal sleep studies could not be predicted based on the presence or absence of pre-morbid conditions such as intraventricular hemorrhage, hypoxic-ischemic encephalopathy, bronchopulmonary dysplasia, or status-post ligation of patent ductus arteriosus. The length of hospital stay after the sleep study was similar in both groups.

### 3.2. Cardio-Respiratory Events and Relation to Time of Study (Epoch A vs. Epoch B) 

The event frequency for grouped data of all recorded events, including central, obstructive, and mixed apnea episodes, periodic breathing, desaturation, and bradycardia episodes, were similar in epoch A (Afternoon 12 noon to 10 pm) and epoch B (Bedtime 10 pm to 8 am) and there was no significant pattern of difference with the aggregate 20-h data.

### 3.3. Cardio-Respiratory Event Differences Between Normal and Abnormal Sleep Studies: (Table 2)

The most consistent differences in the findings of sleep studies that were normal vs. abnormal were in the total duration of periodic breathing as well as the percent of study time spent in periodic breathing. This was consistent in the epochs or comparing any 10-h epoch vs. the total 20-h study. The reporting threshold for abnormal periodic breathing was >5% of the study time. Total study time with oxygen desaturations on pulse oximetry was similar in the normal and abnormal studies and did not vary by time epoch or by 10 vs. 20 h reporting. There were no consistent findings in the total number of bradycardia episodes based on epoch or 10 vs. 20-h study time. There was wide variation in the duration of bradycardia per episode that reached statistical significance when the total 20-h study time was considered (Table 2).

**Table 2:** Cardiorespiratory events in normal vs. abnormal Multichannel Sleep Studies.

	Epoch A (Afternoon—10 h)			Epoch B (Bedtime—10 h)			Full 20-h Study		
	Normal	Abnormal	*p*-Value	Normal	Abnormal	*p*-Value	Normal	Abnormal	*p*-Value
Patient Number	16	34		19	31		9	41	
Total Periodic Breathing (sec)	908 ± 460	4475 ± 3864	<0.0001	980 ± 554	4628 ± 3993	<0.0001	1744 ± 972	7636 ± 6938	<0.0001
Total Desaturation (sec)	236 ± 278	439 ± 669	0.137	244 ± 310	357 ± 673	0.424	351 ± 292	762 ± 1148	0.050
Desaturation/episode (sec)	10.6 ± 3.2	9.4 ± 3.9	0.253	11.4 ± 8.6	9.4 6.7	0.396	11.7 ± 3.8	10.6 ± 6.0	0.493
Total Bradycardia episodes (n)	0.88 ± 1.5	1.7 ± 2.5	0.184	0.79 ± 1.2	1.19 ± 2.7	0.477	1.22 ± 2.2	2.71 ± 4.3	0.150
Bradycardia/episode (sec)	5.9 ± 10.2	16.3 ± 24.3	0.040	8.4 ± 12.4	10.7 ± 25.1	0.663	8.2 ± 15.6	25.9 ± 36.6	0.028

Data represented as Mean ± SD. Repeated measures ANOVA or paired *t*-tests—*p*-value < 0.05.

### 3.4. Missed Cardio-Respiratory Events in 10-h vs. 20-h Multichannel Sleep Study

Of the 50 sleep studies evaluated, 41 infants had abnormal 20-h-multichannel-sleep studies based on the operational definition, and nine were normal. However, if only the first 10 h of study (epoch A—Afternoon) were evaluated, only 34 infants who had abnormal 20 h studies would have been categorized as having an abnormal study, and seven infants (14% of total) that would show abnormalities later would have been missed. Similarly, if only the later 10-h period of the study (epoch B—Bedtime) were evaluated, 31 infants of the 20 h abnormal studies would have been categorized as having an abnormal study, and ten infants (20% of the total) would show abnormalities in the earlier epoch would have been missed. The high rate of missed events in the 10-h study time was similar in both epochs A and B.

### 3.5. Correlation of Sleep Study Results with Primary Symptoms

**Figure 1:**
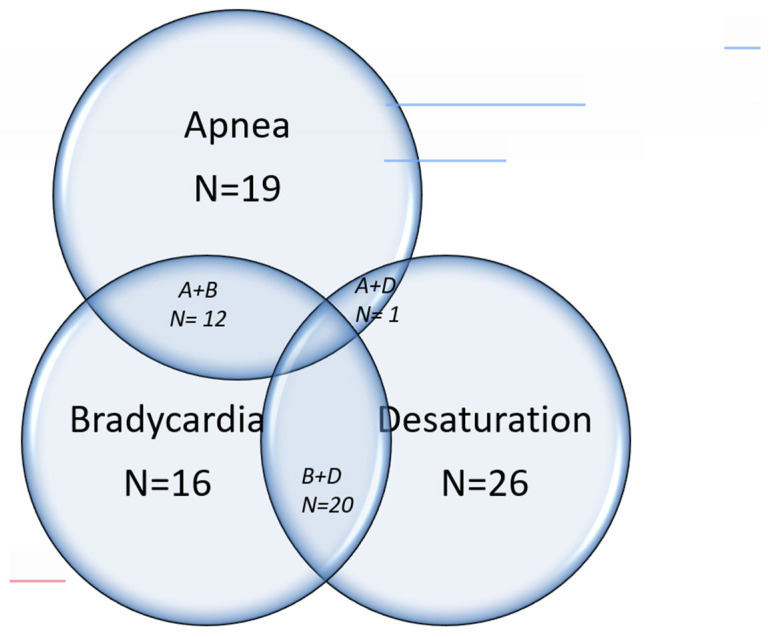
Symptoms that initiated the study: The presenting symptoms of the patients that were studied is shown in the Venn Diagram below. A—Apnea, B—Bradycardia and D—Desaturations.

The common symptoms for which the sleep studies were indicated were immature breathing or apnea, bradycardia, desaturation, or a combination of these symptoms. (Figure 1) The sensitivity, specificity, positive predictive value, and negative predictive value of these symptoms with the final results of the sleep study are shown in Table 3. The positive predictive value was relatively high (>80%) for all the symptoms studied, but sensitivities and specificities were low for individual symptoms or combination of symptoms.

### 3.6. Clinical Utility of the 20-h Multichannel Sleep Study

The study’s results were important in directing clinical management. Twenty-six infants with immature breathing documented as high periodic breathing or central apnea were treated with Caffeine and discharged home on a cardio-respiratory monitor. Twenty-eight infants with obstructive apnea, as the only abnormality, were treated for acid reflux and monitored for clinical resolution of symptoms before discharge. There was an overlap in 15 infants that needed both Caffeine and medications for acid reflux prior to discharge. Infants with normal studies did not need any further treatment, and discharge was expedited.

## 4. Discussion

The role of multichannel sleep studies in evaluating an infant with apnea, bradycardia, and oxygen desaturations is a subject of debate and study [20]. The indications for its use are not well established, and a limited number of NICUs can perform this study. The optimal duration of the study to detect infants with abnormal or normal cardiorespiratory function has not been well characterized. Therefore, our findings from this report will be important in establishing the optimal timing and duration of the sleep study in recovering premature infants.

We have shown that the normal and abnormal studies differ in many aspects, but the most consistent findings were related to abnormalities in periodic breathing and apnea. The changes in bradycardia and desaturation episodes were inconsistent. During the early periods of development of the Multichannel Sleep Study methodology, it was noted that the addition of pulse oximetry did not significantly alter the final results [21]. Even if these outputs do not discriminate between normal and abnormal studies, they are helpful in better characterizing the clinical issue and in designing management strategies [22].

Our study is the first to report that the time of day did not affect the cardiorespiratory events evaluated during the sleep study. All recorded events, including apnea episodes, periodic breathing, desaturation episodes, and bradycardia episodes, were similar in epoch A (Afternoon, 12 noon to 10 pm) and epoch B (Bedtime, 10 pm to 8 am). There were no diurnal variations or cardio-respiratory events that would result in an abnormal study that occurred randomly at different times of the day. Therefore, an epoch A or Afternoon 10-h sleep study would have detected only 34 infants of the 41 abnormal studies, and the epoch B or Bedtime 10-h Multichannel Sleep Study would have detected only 31 infants of the 41 abnormal studies. These findings strengthen the argument that at least 20 h of sleep study evaluation may be needed to comprehensively evaluate cardiorespiratory events in infants in the NICU. A review of the literature showed that variations in findings have been noted in sleep studies done on different day in the same baby, but diurnal variations have not been reported [23].

We did not find any demographic variables that impacted the study results. Comorbid respiratory conditions have been correlated with abnormal studies, but in our study, the presence of respiratory morbidity did not influence study outcomes [24]. The effect of post-menstrual age on cardiorespiratory events could not be evaluated in this study but earlier reports have found a negative correlation between GA at birth and duration of immature breathing at term post-menstrual age [25]. Apnea of prematurity may persist beyond full-term post-menstrual age in infants born at extremely low gestational ages [25].

The common indications for multichannel sleep studies in former premature infants are immature breathing or apnea, bradycardia or desaturations, or a combination thereof. There are no reports in the published literature of the sensitivity or specificity of any one or a combination of symptoms for which multichannel sleep studies are done. In this study, we have demonstrated that in evaluating apnea, bradycardia, and desaturation episodes after term-corrected gestational age, multichannel sleep has a high specificity and PPV but a low sensitivity and NPV. This finding may be important in proper patient selection and interpretation of such studies.

Our report is limited by its relatively small sample size of 50 infants, which may limit the strength of the conclusions that can be drawn. For the same reason, multiple logistic regression analyses of various confounding factors could not be done to evaluate their contribution to the sleep study’s outcome. Selection bias may be another limitation because the study sample was a convenience sample. This was limited as much as possible by taking consecutive studies without exclusions.

Breathing abnormalities may occur randomly over the course of the day, with similar but not identical incidence and frequency between any two 10-h epochs. However, the more extended 20-h evaluation improves the chances of detecting significant breathing abnormalities that could be missed when limiting the study to 10 h. Similar studies from different centers need to confirm these findings before changes to the duration of a neonatal multichannel sleep study can be recommended in former premature infants.

## Figures and Tables

**Table 1:** Demographics and characteristics of infants with normal and abnormal Multichannel Sleep Study.

Variable	Total Patients *N* = 50	Abnormal Multichannel Sleep Study *N* = 41	Normal Multichannel Sleep Study *N* = 9	*p* Value
GA at birth (wk)	32	32.2 ± 5.4	31.4 ± 6.9	0.734
Corrected GA at study (wk)	39.6	39.6 ± 1.7	39.9 ± 1.6	0.668
Corrected GA at discharge (wk)		41 ± 2	41 ± 2	0.363
Birth Weight (gm)	1863 ± 1082	1907 ± 1045	1702 ± 1239	0.655
Weight at test (gm)	2966 ± 452	2985 ± 461	2822 ± 407	0.498
Male Sex	25			0.392
White Race	39	33 (80%)	6 (66%)	0.392
Singleton birth	40	32 (78%)	8 (88%)	0.665
C-section delivery	23	19 (46%)	4 (44%)	1.0
Inborn	15	12 (29%)	3 (33%)	1.0
Co-morbidity present Y/N				
IVH	4	2 (4%)	2 (22%)	0.144
s/p PDA ligation	1	1 (2%)	0 (0%)	1.0
HIE	1	1 (2%)	0 (0%)	1.0
BPD	17	14 (34%)	3 (33%)	1.0
LOS after Multichannel Sleep Study (d)		9 ± 9	14 ± 14	0.331
Total LOS (d)		45 ± 49	37 ±35	0.541

GA—Gestational age; LOS—Length of Stay. Fisher’s Exact test (2-sided) was done for nominal variables. An unpaired *t*-test was done for numerical variables assuming unequal variance in the sample.

**Table 3:** The study’s results correlated to the symptoms preceding the study, and sensitivity, specificity, PPV, and NPV were calculated.

Symptom for Which the Study Was Done		Sensitivity	Specificity	PPV	NPV
Apnea (A)	***N*** = 19	41%	78%	89%	23%
Bradycardia (B)	***N*** = 16	32%	67%	81%	18%
Desaturations (D)	***N*** = 26	51%	44%	81%	17%
A & B	***N*** = 12	27%	89%	92%	21%
B & D	***N*** = 20	39%	56%	80%	17%
A or B or D	***N*** = 38	78%	33%	84%	25%

Apnea, B—Bradycardia and D—Desaturations. The numbers do not add up to 50 because some infants had multiple symptoms. To distinguish the Boolean ‘AND’ ‘OR’ relationships, the bottom row with ‘OR’ relationship has been highlighted. The rest are ‘AND’ relationships or isolated symptoms. A & D combination were insufficient for a meaningful analysis.

## Data Availability

Data are contained within the article. No new data were created or analyzed in this study.
